# A question of morals? The role of moral identity in support of the youth climate movement Fridays4Future

**DOI:** 10.1371/journal.pone.0248353

**Published:** 2021-03-25

**Authors:** Antonia Misch, Susanne Kristen-Antonow, Markus Paulus

**Affiliations:** Department of Psychology, Ludwig-Maximilians-Universität München, Munich, Germany; Middlesex University, UNITED KINGDOM

## Abstract

In the past year, an unprecedented climate movement has risen among European youth, so-called "Fridays4Future" (F4F). Thousands of pupils skip school every Friday to protest for better climate politics. The public debate on the protests contains highly mixed reactions, including praise as well as condemnation. Recent theoretical accounts propose that people’s engagement in community service and actions towards a greater good could be related to their moral identity. Moral identity (MI) is defined as the extent to which being moral is important to the personal identity. The current preregistered study investigates the link between moral identity and participants’ support for F4F in an online survey (N = 537). Results confirm the association between participants’ moral identity and their support for F4F, with the internalization scale predicting passive forms of support and the symbolization scale predicting active forms of support. Additionally, risk perception was found to play an important role. Thus, this study confirms the role of moral identity in people’s pro-environmental engagement and offers new insights in the context of an important and timely issue.

## Introduction

2019 has been the year in which thousands of young people flooded the streets and demanded adequate political action to tackle the climate crisis. Inspired by 15-year-old Swedish Greta Thunberg, who stopped going to school every Friday in order to protest against the insufficient political reactions to the impeding climate crisis, thousands of pupils joined the protests over the next months and formed a unique and unprecedented social movement, called “Fridays for Future” (F4F). Public reactions to the strike have been mixed, from highly supportive to very aggressive. Even people who generally agree with the general demands of F4F think that F4F’s forms of actions (e.g., skipping school, blocking the main traffic knots of the big cities every week) are too radical and obstruct daily business of innocent citizens, and 21% of the general adult population indicate no sympathy at all with the protests [1]. On the other hand, 58% indicate a general feeling of sympathy for the movement, with younger people (under age 30) being more likely to show support than older people (older than 60 years) (66% vs. 56%). A good number of adults even joined the protests and several new adult groups have been founded to support F4F (e.g., “Parents for Future", "Together for Future", “Scientists for Future”).

What are the underlying psychological mechanisms that determine the level of support for an environmental movement like F4F in the adult population? While many models aim to explain when and why people engage in environmental behavior (most of them revealing mixed results, see e.g., [2] for an overview), the intensity and polarization of the debate about F4F suggests that it taps into fundamental values that are deeply engrained in humans’ psychology. A surprisingly high number of moralizing terms are used in this debate [3], suggesting that moral issues might lie at the center of this debate. However, despite cumulating evidence pointing to a leading role of moral motivations in the context of pro-environmental behavior [4–8], we are still far away from understanding the role of moral considerations in this context (e.g., [9]).

In the current paper we therefore investigate the extent to which a person’s moral identity—defined as the extent to which being a moral person is a central aspect of the person’s identity [10]—is associated with their support for the youth climate movement F4F. This research is not only unique and timely, but also offers valuable insights into the underlying psychological mechanisms that foster support for a rather polarizing social movement. Unraveling this relationship will help us to better understand the current political climate in our society, as well as provide more general insights into the underlying motivations behind political engagement with regard to environmental issues.

### Moral identity

Moral Identity (MI), also called the Moral Self Concept, is defined as the extent to which being a moral person is a central aspect of the person’s identity [11]. In other words, it describes whether a number of desirable moral traits (e.g., such as being fair, caring, kind) are seen as important to oneself as a person. The moral traits are conceptualized in a schematic network of several interconnected moral traits, and activating one of these traits should consequently activate all other traits involved in the whole moral self-concept [10]. Thus, moral identity can be assessed by asking participants to first mentalize a person who possesses a set of moral traits (schema activation) and to subsequently answer questions regarding the extent to which being a person like that is important to themselves. Importantly, the specific content of the moral identity can vary between people, so that individuals differ in the importance they assign to different moral traits [12, 15]. However, a certain set of moral traits seems to be commonly shared among many people’s moral identity, such as justice, honesty, benevolence, or reliability [10, 12, 17].

The construct of MI was developed with the purpose of explaining the link between moral judgment and actual moral behavior—a gap that has been apparent in other theoretical accounts on morality, especially accounts that focus on people’s judgements [13–18]. The intention-behavior gap is widely known in all kinds of domains, and also when it comes to pro-environmental behavior [19, 20] (sometimes even to the extent that people who care more about environmental issues are less likely to request information on the environmental impact of products when making purchasing decisions; most likely to avoid negative feelings [21]). MI, in contrast, might bypass this problem as the natural need for self-consistency should motivate people to act according to their self-identity [15]. And indeed, studies using the MI framework suggest that MI is a good predictor for actual behavior: For example, MI has been significantly associated with moral behavior in general, such as prosocial behavior, ethical behavior, civic engagement, and avoidance of antisocial behavior on a large number of studies [10, 11, 16, 18, 22–24]. It also has been found to be linked to lower ingroup bias in intergroup resource allocations [25–27], feelings of guilt when not acting in a moral way [28] as well as willingness to sacrifice time for a prosocial cause [26]. Finally, MI has also been associated with political and civic engagement in general [29, 30], as well as to pro-environmental behavior such as ethical consumer choices [5, 31, 32]. Thus, numerous studies have confirmed the link between a person’s MI and their actual prosocial behavior.

#### Moral identity and pro-environmental engagement

There is good reason to assume that moral identity might be a particularly interesting and important component to explain people’s support of the youth climate movement F4F. As an unprecedented complex collective action problem, the climate change issue poses an incredible challenge to people’s moral evaluation and behavior. All humans contribute to climate change, but especially those living in rich and industrialized countries. Even though the majority of humans will be affected by the consequences of climate change at some point, the victims who will suffer most (or who are already suffering) are the least advantaged and those living in developing countries [33, 34]. Thus, the psychological distance (i.e., the extent to which people or objects “are not directly present in the direct experience of reality” [35]) between those contributing most to the problem and those who will suffer most from its consequences is quite large (e.g., [36]). For people living in advantaged areas, it is easy to disengage from the consequences for others who live far away (spatial psychological distance), or for consequences that will happen in the future (temporal psychological distance). Multiple studies confirm the negative association between psychological distance and the intention to act on climate change ([36–39] but see [40] for a critical review). A moral viewpoint might offer a solution for the problem of psychological distance, as morality is traditionally associated with the stance of impartiality (e.g., [41–43]). In line with this, previous research has found that people with a high moral identity seem to have a wider "circle of moral regard", that is, they feel more connected to people who do not belong to their immediate group members and also show stronger moral concerns for others who are not close to them [25, 27, 44]. Therefore, we assume that people with a high moral identity should also show more moral concern about the consequences of climate change than people with a low moral identity.

The second reason for why moral identity might be particularly powerful in explaining environmental engagement is the high level of responsibility diffusion in the climate crisis. Diffusion of responsibility describes a phenomenon in which people fail to engage in action when others share this responsibility, too [45]. The climate crisis is an extreme example of diffusion of responsibility, as all people are contributing to it, but no individual contribution alone is enough to create tangible damage [46]. Because of their extended circle of moral regard, people with a high moral identity should not only feel responsible for the consequences of their own behavior but also for the behavior of others. Recent research confirmed the link between moral identity and increased levels of responsibility in people’s green consumption tendency [32]. We thus believe that these increased level of responsibility in participants with a high moral identity might also foster the motivation to support the youth climate movement.

#### Moral considerations in the context of fridays for future

Up to date, research on the movement of F4F is still scarce. The little research that is available mostly consists of polls describing demographics and descriptive data [47, 48]. However, those results underpin the relevance of the present research: For example, Wahlstroem and colleagues [48] found that many adult participants reported a moral obligation for their engagement in the movement. Another poll shows that many older citizens support the youth climate movement [49]. Thus, previous research shows that moral considerations play a role in participants’ engagement in F4F.

However, political movements can only be successful when their notions are supported by the general population [50]. Thus, in the current study we focused on moral identity in the broader population (i.e., adults in Germany), rather than on the activists themselves (see [51] for an overview).

### Aim of the present study

The aim of the current preregistered study was to investigate the extent to which moral identity is associated to adults’ support of the youth climate movement F4F (https://aspredicted.org/blind.php?x=js7cy5). We therefore administered a survey which assessed participants’ moral identity, measured with the Self-Importance of Moral Identity Questionnaire (SMI-Q, [10]), as well as their self-reported levels of support of said movement. We expected to find a positive relationship between participants’ MI and their levels of support (H1). The SMI-Q consists of two distinct dimensions which are proposed to motivate moral behavior via two distinct pathways: The internalization scale describes the actual centrality of morality to a person’s inner self-concept. Because people generally hold a strong desire to act in consistency with their inner self-concept, people who score high on the internalization scale should also show a stronger motivation to act morally [52]. The symbolization scale, in contrast, describes how important it is to be recognized as a moral person by other people. Thus, people who score high on the symbolization scale seek to verify their identity through the reflected appraisal of others, for example through the use of symbols or through public actions [52]. Both scales have been found to be moderately correlated (e.g., [10, 25]).

As main dependent variable we assessed people’s support for F4F with a short scale we developed. In line with previous research on related issues (Civic Engagement Scale, [53]) and to get a more thorough picture of support, we differentiated two subscales. The scale of *Passive Support* was designed to measure participants’ personal beliefs and attitudes about F4F, whereas the scale of *Active Support* was designed to measure participants’ actual behavior with which they had supported F4F in the past (e.g., joining the demonstrations, donating money). We hypothesized that both scales would be associated with participants’ moral identity, but expected that the symbolization scale might be a slightly better predictor for actual behavior than for passive support [14, 24, 52], whereas the internalization scale might be better in predicting participants’ passive support [14, 52] (H2).

However, in order for moral identity to come into play the issue at hand has to have some importance of moral relevance for the individual. For example, people who do not believe that climate change poses a risk to the next generation might not feel the need to support F4F, independent of their moral identity. Risk perception—the extent to which participants perceive climate change as a threat—might therefore play a moderating role in the relationship between moral identity and support for F4F. The importance of threat perception for participants’ motivation to engage in pro-environmental behavior has been demonstrated in previous research [54–56]. We therefore additionally assessed whether participants perceived the consequences of climate change as threatening (*risk perception*). Furthermore, to gain a clearer picture, we also assessed whether they engaged in other forms of pro-environmental behavior (*engagement in other pro-environmental organizations*; *personal pro-environmental behavior*). Finally, we added a Social Desirability Scale to control for socially desired response patterns [57].

## Method

### Participants

A power analysis was conducted based on a medium effect size of *r* = 22 [23], *α* = 0.05 and power of 0.95 and yielded a minimum sample size of 219. Participants were invited to participate in the online survey via e-mail link sent out by students who helped on this study as part of a classroom project. Thus, participants were recruited among students’ families, friends, work colleagues and hobby clubs (e.g., sports associations), including urban and rural areas. As we decided not to exclude any surplus participants, the final sample consisted of 546 German participants (175 male) between 16 and 90 years of age (*M* = 33.5; *SD* = 16) (1 person had to be excluded because they were too young to legally consent to participate). This research was approved by the Ethical Committee of LMU Munich, Department of Psychology.

### Measures

All measures were administered in an online survey, for which participants gave their consent to participate in anonymously. The activities of F4F had been widely covered in German public media and social media over the past months, and thus we had no doubt that all participants were well informed about this subject. However, to ensure that all participants were aware of some important facts about the movement, they read a short description of the youth climate movement "Fridays for Future" (see S1 Appendix) at the beginning of the survey. They were then asked to answer a number of questions regarding their support (*active* and *passive support*), additional variables (*engagement in other pro-environmental organizations*, *personal pro-environmental behavior*, *risk perception*), *social desirability*, distractor variable (*scale of self-responsibility*), and *moral identity*. All participants answered the questions in the same fixed order so that moral identity was assessed last to avoid priming participants with moral concepts beforehand, as previous research has shown that the moral identity survey can alter participants’ subsequent responses [58]). Additional variables and distractor variables were assessed in between the two measures support and moral identity. In what follows, however, variables are sorted by importance for our research question.

#### Support

The scale measuring participants’ support for F4F consisted of two short subscales (*passive support*, *active support*). Overall scale consistency was good with Cronbach’s α = .84.

*Passive support*. Participants read 6 statements (e.g., "I like the movement F4F ", "I approve of the weekly protests, even though they block the traffic in the big cities") and were asked to indicate their agreement to each on a 7-point Likert scale (1 - "I do not agree at all" to 7 "I completely agree"). Because of the novelty and recency of the youth climate movement, we could not rely on previous instruments. Items were developed in classroom discussions based on public perception (press and media statements) of Fridays for Future, containing items measuring positive affection in general (e.g., "I like the F4F movement"), justification of radical measures (e.g., "Although they stay away from school, the students of F4F act right."), and willingness to support the movement (e.g., "I would like to support F4F"). Scale consistency was excellent, with Cronbach’s α = 93. (As this scale was not based on existing scales, we ran an exploratory factor analysis. Ordinary Least Squared/Minres parallel analysis recommended a two-factor solution. An Oblimin-rotation revealed loadings >0.3 on one factor only, suggesting that a one-factor solution is appropriate with sums of squared loadings of 4.14.)

*Active support*. A number of questions assessed participants’ behavior in support of F4F, including e.g., attendance of protests organized by F4F, signing of petitions, and donations. The items were based on the Activism Scale of Seguin, Pelletier, and Hunsley [59] and adapted to fit our context.

Three items were dropped (amount of donations, strength of active involvement in partner organization, other kind of support) due to low variability in responses (e.g., only 6% of participants had indicated that they had donated money in the past). Response format varied between questions, see S1 Appendix. Reliability was low with Cronbach’s α = 0.48.

*Moral identity*. We assessed moral identity using the Self-Importance of Moral Identity Questionnaire (SMI-Q) by Aquino and Reed [10]. Overall scale consistency was good with standardized *Cronbach’s α = 0*.*80* (internalization scale: *α* = 0.77; symbolization scale: *α* = 0.71).

#### Additional variables

*Engagement in other pro-environmental organizations*. Participants were asked to rate their engagement in other organizations (e.g., Greenpeace or WWF) in terms of membership, protest, donations, and other activities on a scale from 1 (not at all) to 4 (very much). Scale consistency was fair with *Cronbach’s α* = .74 (*M* = 6.85; *SD* = 2.82).

*Personal pro-environmental behavior*. Participants were asked to indicate their agreement to 5 statements about their own personal pro-environmental behavior (e.g., going to work by car, eating meat every day) on a scale from 1 (not at all) to 4 (very much). Scale consistency was low with *Cronbach’s α* = .6 (*M* = 14.83; *SD* = 3.09).

*Risk perception*. Participants were asked to rate their perceived danger of climate change (“How dramatic do you consider the consequences of climate change?”) on a scale from 1 (not dramatic at all) to 4 (very dramatic).

*Social desirability*. Before assessing participants’ moral identity, we assessed their tendency to respond in a socially desirable way using the 7-item scale by Satow [57]. Scale consistency was low with *Cronbach’s α = 0*.*67*. As distractor items we included 5 items of a scale of self-responsibility [60], which were not of interest and therefore not analyzed.

*Demographics*. Finally, we assessed participants’ age, gender and level of education.

## Results

We used R [61] to analyze standardized and mean-centered data via correlations and linear models. In the linear models, terms were retained when significant or when contained in a higher-order interaction. Assumptions of linear models were tested using the function gvmla [62] and confidence intervals of bootstrapped regression (R = 2000) were added when assumptions were violated. Data and R script is openly available at https://osf.io/chqwa/?view_only=b78488698f4f4eacbf59230cb1e11bbe.

### Preliminary analysis

To gain a first overview, we ran a correlation including the individual subscales of all the measures. Due to several violations of the assumptions (e.g., several variables were not normally distributed), a Spearman correlation was used. It revealed high correlations between several scales: As expected, the subscales for both moral identity (internalization and symbolization) and for support (active support and passive support) were strongly related. Furthermore, we found several correlations between moral identity scales, support scales, risk perception, personal behavior and engagement in other organizations (see Table 1). In contrast, there was only a weak link between social desirability and moral identity scales, and no correlation with any support scale, suggesting that social desirability was not a leading factor in the subjects’ responses.

**Table 1:** Correlation matrix of all variables (Spearman correlation).

	1	2	3	4	5	6	7	8	9
1. Internalization									
2. Symbolization	0.43***								
3. Passive Support	0.21***	0.11**							
4. Active Support	0.09*	0.14**	0.46***						
5. Risk Perception	0.20***	0.05	0.42***	0.24***					
6. Social Desirability	0.09*	0.09*	0.01	0.03	-0.05				
7. Personal Behavior	0.14***	0.11*	0.39***	0.38***	0.37***	0.15***			
8. Other Organization	0.03	0.07	0.28***	0.34***	0.20***	0.05	0.28***		
9. Age	-0.14***	-0.05	-0.14***	-0.19***	-0.20***	0.21***	-0.03	0.08	
10. Gender	0.13**	0.09*	0.13**	0.04	0.19***	0.01	0.24***	0.09*	-0.07

Note

* *p* < .05

** *p* < .01

*** *p* < .001

Descriptive statistics can be found in Table 2.

**Table 2:** Descriptive statistics of all variables.

*Variable*	*Mean*	*SD*	*Min*	*Max*
1. Moral Identity Sum	48.3	9.1	18	70
1a. Internalization	28.2	5.3	7	35
1b. Symbolization	20.2	5.5	5	35
2. Support Sum	33.2	10.3	8	52
2a. Passive Support	30.4	9.7	6	42
2b. Active Support	2.9	1.5	2	10
3. Risk Perception	3.7	0.6	1	4
4. Social Desirability	18.4	3.6	9	28
5. Personal Behavior	14.8	3.1	5	20
6. Other Organization	6.9	2.8	4	16
7. Age	33.5	16.0	16	90

### Moral identity and support for fridays for future

Our main question of interest was whether the strength of the moral identity is linked to the level of support (active and passive combined) for F4F. We found that moral identity (sum score) and the support for F4F (sum score) were significantly correlated (Spearman correlation; *r*(544) = .19, *p* < .001).

To investigate which scale of the moral identity, internalization or symbolization, is a better predictor for support in general, we ran a linear model with both scales as predictors, including also gender and age, as well as social desirability (model 1). The model violated assumptions of linearity, normal distribution and continuous distribution, and therefore we repeated the analysis via bootstrapping. When both scales were added as predictors in the model, the internalization scale was significant (*β* = 0.13, *t*(540) = 2.86, *p* = .004; *bootstrapped 95% CI* [0.03, 0.23]), whereas the symbolization scale was not (*p* = .46; *bootstrapped 95% CI* [-0.07, 0.14]). Additionally, we found main effects of gender (*β* = -.27, *t*(540) = -2.96, *p* = .003; *bootstrapped 95% CI* [-0.46, -0.08]), suggesting that females were more supportive of F4F than males, and of age (*β* = -0.10, *t*(540) = -2.22, *p* = .027; *bootstrapped 95% CI*[-0.19, -0.01]), suggesting that younger participants were more supportive than older participants. Social desirability had no effect, suggesting that participants’ responses regarding their levels of support were not guided by their desire to appear in a positive light (*p* = .65) (see Table B.1 in S2 Appendix).

#### Passive support

B ased on previous research suggesting that the internalization scale might be a better predictor for attitudes, and the symbolization scale for actual behavior, we ran separate models for both types of support, including both moral identity scales (model 2).

**Fig 1:**
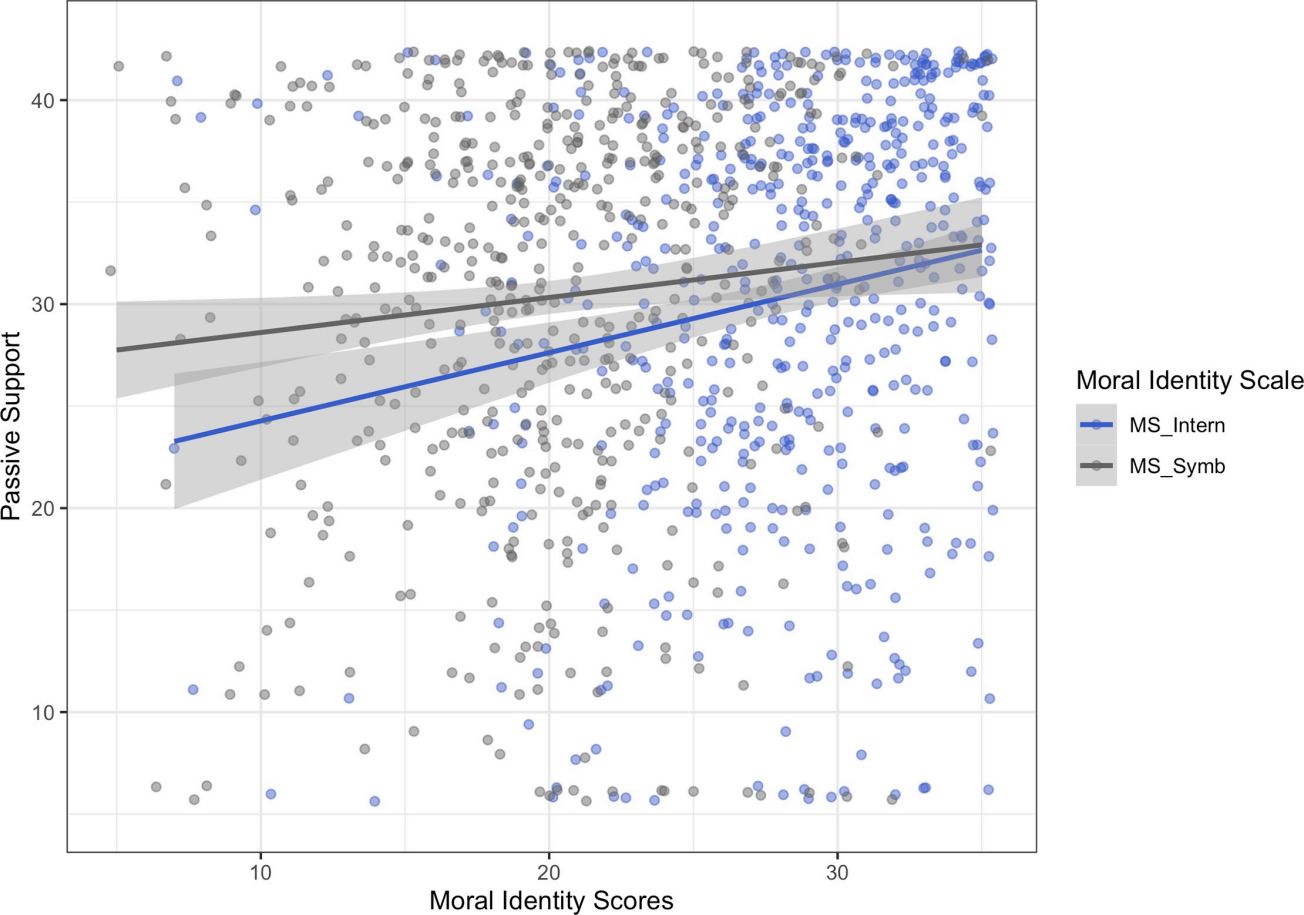
Linear regression: Symbolization scale and internalization scale predicting passive support of F4F, with 95% confidence band.

The model predicting passive support violated assumptions of linearity and normal distribution, therefore we repeated the analysis via bootstrapping. With both scales in the linear regression predicting people’s passive support (which reflect people’s attitudes), we found that the internalization scale was a significant predictor (*β* = 0.15, *t*(541) = 3.15, *p* = .002; *bootstrapped 95% CI* [0.04, 0.26]), whereas the symbolization scale was not (*p* = .71; *bootstrapped 95% CI* [-0.08, 0.12]) (Fig 1). Additionally, we again found a main effect of gender (*β* = -0.29, *t*(541) = -3.2, *p* = .001; *bootstrapped 95% CI* [-0.49, -0.11]), suggesting that females showed higher levels of passive support than males. A marginal effect of age was not significant in the bootstrapped model (*β* = -0.08, *t*(541) = -1.78, *p =* .077; *bootstrapped 95% CI* [-0.16, 0.01]). Social desirability had no effect (*p* = .79, *bootstrapped 95% CI* [-0.07, 0.11]), (see Table B.2 in S2 Appendix).

#### Active support

**Fig 2:**
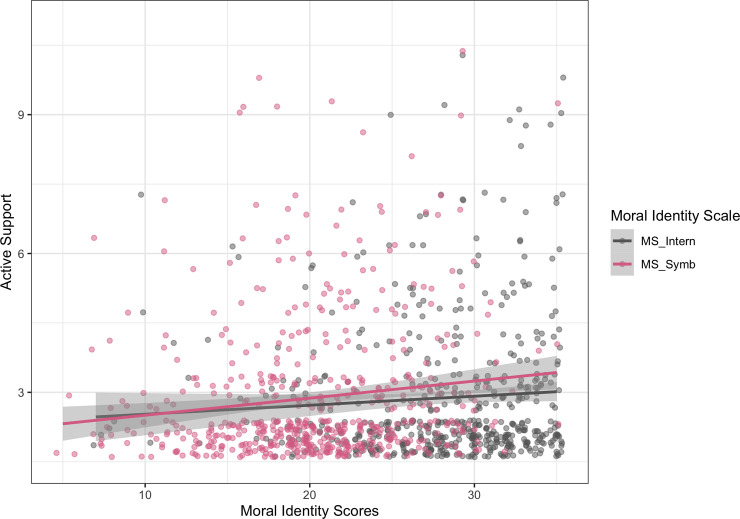
Linear regression: Symbolization scale and internalization scale predicting active support of F4F, with 95% confidence band.

The model (model 3) violated assumptions of linearity, normal distribution and heteroscedasticity, therefore we repeated the analysis via bootstrapping. We found that the symbolization scale was a significant predictor for active support (*β* = 0.134, *t*(541) = 2.78, *p* = .006; *bootstrapped 95% CI* [0.03, 0.23]), whereas the internalization scale was not (*p* = .74; *bootstrapped 95% CI* [-0.12, 0.07]) (Fig 2). Additionally, age was significant (*β* = -0.17, *t*(541) = 3.81, *p* < .001; *bootstrapped 95% CI* [-0.26, -0.08]), suggesting that active support decreased with age. No effect was found for gender (*p* = .83, *bootstrapped 95% CI* [-0.15, 0.2]), nor for social desirability (*p* = .17, *bootstrapped 95% CI* [-0.02, 0.14]) (see Table B.3 in S2 Appendix).

### Moral identity and risk perception

**Fig 3:**
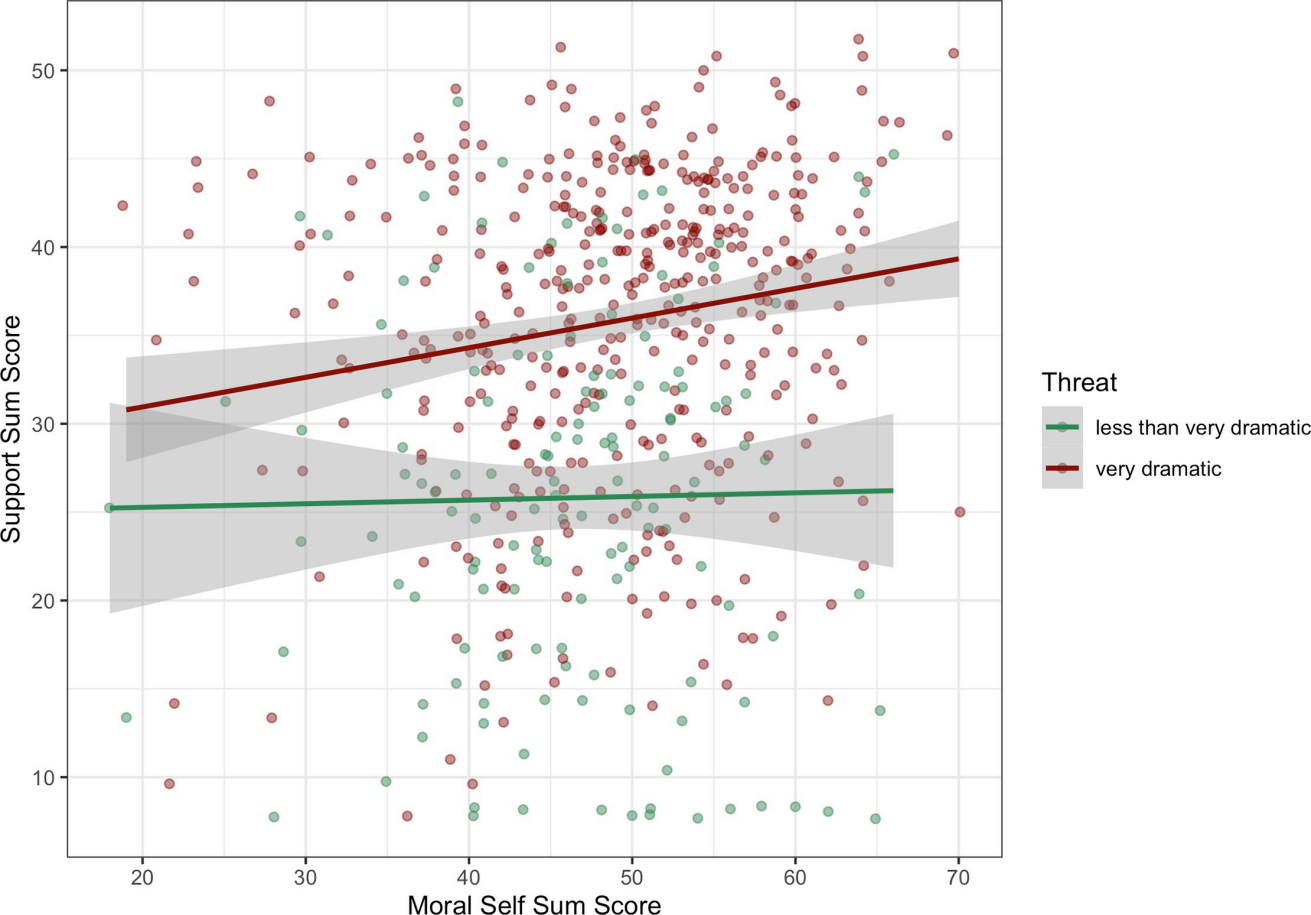
Linear regression: Support predicted by moral identity and risk perception. *Note*: for ease of presentation, this figure deviates from the regression model: There was no interaction between moral identity and risk perception in the bootstrapped model, but we wanted to represent both variables in one graph. In addition, we collapsed all participants who perceived climate change as less than very dramatic into one group (represented by red). Green represents participants who perceive climate change as very dramatic. The regression model contained the numerical values (1–4).

In order to investigate the role of risk perception in participants’ willingness to support F4F, we ran another linear model (model 4), containing moral identity (sum score), risk perception, age and gender as predictors and support (sum score) as response variable. These variables were entered as interaction terms, in order to allow for the detection of potential moderation effects. We hypothesized that only people with an understanding of the risks of climate change would feel the need to act in line with their moral identity in this particular context. This effect might be stronger for younger people who are more likely to suffer from the consequences in the coming decades, and therefore we entered age as well. Finally, gender was included as an interaction term as this movement has been described as a particular female movement, and thus might spark more support among women. In addition, we added social desirability. Besides main effects of moral identity (*β* = .09, *t*(536) = 2.03, *p* = .022) and risk perception (*β* = .47, *t*(536) = 11.53, *p* < .001), we found a three-way interaction of moral identity, age and risk perception (*β* = -.08, *t*(536) = -2.02, *p* = .043), suggesting that the association between moral identity and support was stronger with increasing age and increasing risk perception. The model violated the assumptions of linearity and normal distribution, therefore we repeated analysis via bootstrapping. The bootstrapped results indicated that the three-way interaction was not significant (*bootstrapped 95% CI* [-0.17, 0.01]), and was therefore dropped. The final model revealed no other significant interaction (*p*s>.19), but significant main effects of perceived risk (*β* = 0.45, *t*(540) = 11.70, *p* < .001; *bootstrapped 95% CI* [0.36, 0.53]) and moral identity (*β* = 0.11, *t*(540) = 2.77, *p* = .006; *bootstrapped 95% CI* [0.03, 0.20]), suggesting that the perception of climate change as a threat is even a stronger predictor for peoples support of F4F than moral identity (Fig 3). In this model, age, gender and social desirability were not significant in predicting people’s support of F4F (all *p*s>.16), (see Table B.4 in S2 Appendix).

## Discussion

The aim of this study was to explore the underlying psychological aspects that explain people’s support for climate activism. More specifically, we investigated the extent to which moral identity is linked to the support of the youth climate movement Fridays for Future (F4F). Results reveal that both are linked, as indicated by the correlation between Moral Identity and Support, wherein the internalization scale seems to be a stronger predictor than the symbolization scale. Thus, our research dovetails nicely with previous findings indicating that moral identity motivates prosocial behavior in general ([10, 11, 16, 18, 24], see meta-analysis by Hertz & Krettenauer [23]), and more specifically, pro-environmental behavior [5, 31, 32]. It also extends previous research by showing that moral identity can help to explain the extent to which people show support towards a pro-environmental activist movement and highlights the psychological factors that might be related to people’s support of F4F.

While it was not the primary goal of our study to disentangle the underlying psychological processes of passive versus active support, more fine-grained analyses of our study do offer valuable insights into the distinct underlying motivations of the two forms of support for F4F. While both the internalization and symbolization scale of the MI are correlated with both forms of support, the regression analyses show that internalization seems to be a better predictor for passive support than the symbolization scale, and vice versa. This finding supports theoretical accounts suggesting that two different underlying motivations are determining the two distinct dimensions of moral identity: Based on Blasi’s theoretical account [15], Winterich and colleagues [52] propose that people high (vs. low) in internalization of MI should be motivated to act accordingly primarily through their desire for self-consistency, regardless of whether their actions are visible to the public. In contrast, people high in symbolization should be primarily motivated to verify their self through the reflected appraisal of others ([52], based on [15]), and thus should be especially motivated to act in accordance with their MI when their actions can be seen by others. While we did not differentiate between visible and invisible forms of support of F4F in our study, the passive forms of support are inherently more private, as they reflect the participants’ attitudes towards the movement. Our active support scale, in contrast, contains items that reflect publicly visible acts of support, such as participating in demonstrations and signing petitions. Thus, our results support the notions that the two dimensions of MI spark moral behavior through different motivational pathways. However, one caveat with respect to these findings is the low internal consistency of the active support scale, and thus they should be regarded with some caution. Due to the recency of the formation of F4F at the timepoint of the study, participants have had only little opportunities (i.e., a few months) to engage actively with this movement, reducing our choice of items dramatically (our final scale consisted only of four items, two of which required only a yes/no response). Furthermore, the behaviors we assessed are behaviors that are typically only displayed by a small percentage of the population (e.g., only 8% percent of our sample reported to have participated in more than one protest activity, and 82% percent have never attended), and many participants who might have been willing to attend protests might not have been able to leave their job on a Friday at noon, thus reducing overall variability of the responses in that scale even more. Despite this limitation, the items in itself and their relationship with the other variables are nevertheless informative for the matter at hand, and provide a good starting point for future research.

In order to get a better picture of other potential factors that are linked to a supportive standpoint, we also assessed a number of additional variables. The correlation revealed that participants who were supportive towards F4F reported higher rates of pro-environmental behavior in their personal life and more engagement in other pro-environmental organizations. Furthermore, a negative association with age confirmed that younger participants were more supportive of F4F. Finally, we found a positive association between risk perception and support for F4F, which we also investigated in a regression analysis. Risk perception—the extent to which participants evaluated climate change as a threat–was found to be even a stronger predictor of support for F4F than moral identity. Our research therefore highlights the importance of risk perception as a motivator to support the youth climate movement. On a broader picture, this study thus aligns with research finding that risk perception is a general motivator for pro-environmental behavior (e.g., [55, 56]).

Furthermore, we investigated potential effects based on age and gender. We found that women showed generally higher levels of support for F4F than males. This is in line with research demonstrating that women often show higher levels of pro-environmental concern and behavior [63–67]. In addition, the movement F4F in itself, including the leaders, is composed of more female than male students [47, 48]. However, when looking at actual behavior (active support) only, our study did not reveal any differences based on gender. This is in contrast with a previous meta-analysis showing that gender differences were stronger regarding pro-environmental behavior than regarding attitudes [67]. One potential explanation for this peculiar finding is that as in most studies relying on unpaid volunteer participants, our sample might have attracted more people who were already more prosocial and showed a higher interest for the issue of climate change. Thus, the fact that female participants outweighed male participants by large in our sample might already reflect the female preponderance in the support for environmental values. Ideally, future research should utilize different recruitment strategies to avoid bias due to self-selection. Another potential reason pertains to the specificity of the behaviors we assessed in the active support scale. While previous research has looked at pro-environmental behavior in general, including personal behavior, we looked specifically at active forms of support for F4F, rather than general forms of pro-environmental behavior.

It is interesting to note that gender differences disappeared when we added risk perception into the model, hinting at a potential mediating role of risk perception on gender differences in pro-environmental engagement. Previously, gender differences in pro-environmental attitudes and behavior have often been explained in light of gender differences in socialization regarding other-orientation and social responsibility [67]. Our study opens up another possibility, in that gender differences in pro-environmental behavior and activism might be explained by differences in risk perception (e.g., [68, 69]), an avenue which deserves further attention in future research.

One limitation of our study concerns the fact that it does not fully represent the German population. However, we recruited participants from different areas, including rural areas and diverse backgrounds, and thus our study sample is more representative of the general population than the samples that are typically recruited in University settings. Another limitation refers to the fact that as with all correlational and cross-sectional research, conclusions regarding the direction of the relationship between moral identity and the support for F4F can only be drawn with caution, and interpretations are based on previous research and theoretical knowledge. In addition, in order to avoid priming effects [58], we did not randomize the order in which the measures were assessed, thereby allowing for potential sequencing effects. Experimental manipulations and longitudinal research might provide a more thorough picture in the future. However, given the unpredictability of how events unfold in the real world, this might be hard to accomplish. Studying the link between moral identity and the support of a real existing political movement—rather than a hypothetical one–is one of the main assets of the present study.

Taken together, the current research contributes to a better understanding of aspects of the psychological framework that motivate people to support the pro-environmental movement F4F. By investigating the extent to which moral identity influences people’s attitudes and behavior regarding F4F, our study provided evidence for the centrality and leading role that moral considerations play for civic and political engagement in human societies.

## Supporting information

S1 Appendix (DOCX)

S2 Appendix (DOCX)

## References

[1] Koos S., & Naumann E. (2019). Vom Klimastreik zur Klimapolitik: Die gesellschaftliche Unterstützung der „Fridays for Future “-Bewegung und ihrer Ziele: Forschungsbericht. Working paper, derived from: https://kops.uni-konstanz.de/handle/123456789/46901

[2] Dono J, Webb J, Richardson B. The relationship between environmental activism, pro-environmental behaviour and social identity. Journal of Environmental Psychology. 2010;30(2):178–86. 10.1016/j.jenvp.2009.11.006

[3] Reinhardt S. Fridays For Future–Moral und Politik gehören zusammen. GWP–Gesellschaft, Wirtschaft, Politik. 2019;68(2):159–62. 10.3224/gwp.v68i2.01 German.

[4] Feinberg M, Willer R. The Moral Roots of Environmental Attitudes. Psychological Science. 2013;24(1):56–62. 10.1177/0956797612449177 23228937

[5] Jia F, Soucie K, Alisat S, Curtin D, Pratt M. Are environmental issues moral issues? Moral identity in relation to protecting the natural world. Journal of Environmental Psychology. 2017;(52):104–13. 10.1016/j.jenvp.2017.06.004

[6] Knez I. Is Climate Change a Moral Issue? Effects of Egoism and Altruism on Pro-Environmental Behavior. Current Urban Studies. 2016;4(2):157–74. 10.4236/cus.2016.42012

[7] Bamberg S., & Möser G. (2007). Twenty years after Hines, Hungerford, and Tomera: A new meta-analysis of psycho-social determinants of pro-environmental behaviour. Journal of environmental psychology, 27(1), 14–25.

[8] Van der Werff E, Steg L, Keizer K. It is a moral issue: The relationship between environmental self-identity, obligation-based intrinsic motivation and pro-environmental behaviour. Global Environmental Change. 2013;23(5):1258–65. 10.1016/j.gloenvcha.2013.07.018

[9] Moore KD, Nelson MP. Moving toward a global moral consensus on environmental action. In: Starke L, editor. State of the World 2013: is sustainability still possible? Washington, D.C.: Island Press; 2013. p. 225–33.

[10] Aquino K, Reed A II. The self-importance of moral identity. Journal of Personality and Social Psychology. 2002;83(6):1423–40. 10.1037//0022-3514.83.6.1423 12500822

[11] Hardy SA, Carlo G. Moral identity: What is it, how does it develop, and is it linked to moral action? Child Development Perspectives. 2011;5(3):212–8. 10.1111/j.1750-8606.2011.00189.x

[12] Walker LJ, Hennig KH. Differing Conceptions of Moral Exemplarity: Just, Brave, and Caring. Journal of Personality and Social Psychology. 2004;86(4):629–47. 10.1037/0022-3514.86.4.629 15053710

[13] Hardy S. A., Walker L. J., Olsen J. A., Skalski J. E., & Basinger J. C. (2011). Adolescent naturalistic conceptions of moral maturity. Social Development, 20(3), 562–586.

[14] Lapsley D. K., & Lasky B. (2001). Prototypic moral character. Identity: An International Journal of Theory and Research, 1(4), 345–363.

[15] Blasi A. Bridging moral cognition and moral action: A critical review of the literature. Psychological Bulletin. 1980;88(1):1–45. 10.1037/0033-2909.88.1.1

[16] Blasi A. Moral Cognition and Moral Action. Developmental Review. 1983;3(2):178–210. 10.1016/0273-2297(83)90029-1

[17] Hardy SA, Carlo G. Identity as a source of moral motivation. Human Development. 2005,48(4):232–56. 10.1159/000086859

[18] Hart D, Fegley S. Prosocial Behavior and Caring in Adolescence: Relations to Self‐Understanding and Social Judgment. Child Development. 1995;66(5):1346–59. 10.1111/j.1467-8624.1995.tb00939.x 7555220

[19] Grimmer M, Miles MP. With the best of intentions: a large sample test of the intention-behaviour gap in pro-environmental consumer behaviour. International Journal of Consumer Studies. 2016;41(1):2–10. 10.1111/ijcs.12290

[20] Juvan E, Dolnicar S. The attitude-behaviour gap in sustainable tourism. Annals of Tourism Research. 2014;(48):76–95. 10.1016/j.annals.2014.05.012

[21] Ehrich KR, Irwin JR. Willful ignorance in the request for product attribute information. Journal of Marketing Research. 2005;42(3):266–77. 10.1509/jmkr.2005.42.3.266

[22] Christner N, Pletti C, Paulus M. Emotion understanding and the moral self-concept as motivators of prosocial behavior in middle childhood. Cognitive Development. 2020;55:100893. 10.1016/j.cogdev.2020.100893

[23] Hertz SG, Krettenauer T. Does moral identity effectively predict moral behavior?: A meta-analysis. Review of General Psychology. 2016;20(2):129–40. 10.1037/gpr0000062

[24] Sunil S, Verma SK. Moral Identity and Its Links to Ethical Ideology and Civic Engagement. Journal of Human Values. 2018;24(2):73–82. 10.1177/0971685818754547

[25] Reed A II, Aquino KF. Moral Identity and the Expanding Circle of Moral Regard Toward Out-Groups. Journal of Personality and Social Psychology. 2003;84(6):1270–86. 10.1037/0022-3514.84.6.1270 12793589

[26] Reed A II, Kay A, Finnel S, Aquino K, Levy E. I don’t want the money, I just want your time: How moral identity overcomes the aversion to giving time to prosocial causes. Journal of personality and social psychology. 2016;110(3):215. 10.1037/pspp0000058 26523999

[27] Winterich KP, Mittal V, Ross WT. Donation Behavior toward In-Groups and Out-Groups: The Role of Gender and Moral Identity. Journal of Consumer Research. 2009;36(2):199–214. 10.1086/596720

[28] Stets JE, Carter MJ. A theory of the self for the sociology of morality. American Sociological Review. 2012;77(1):120–40. 10.1177/0003122411433762

[29] Porter TJ. Moral and political identity and civic involvement in adolescents. Journal of Moral Education. 2013;42(2):239–55. 10.1080/03057240.2012.761133

[30] Pratt MW, Hunsberger B, Pancer SM, Alisat S. A Longitudinal Analysis of Personal Values Socialization: Correlates of a Moral Self-Ideal in Late Adolescence. Social Development. 2003;12(4):563–85. 10.1111/1467-9507.00249

[31] Chowdhury RMMI, Fernando M. The Relationships of Empathy, Moral Identity and Cynicism with Consumers’ Ethical Beliefs: The Mediating Role of Moral Disengagement. Journal of Business Ethics. 2014;124(4):677–94. 10.1007/s10551-013-1896-7

[32] Wu B, Yang Z. The impact of moral identity on consumers’ green consumption tendency: The role of perceived responsibility for environmental damage. Journal of Environmental Psychology. 2018;(59):74–84. 10.1016/j.jenvp.2018.08.011

[33] Samson J, Berteaux D, Mcgill BJ, Humphries MM. Geographic disparities and moral hazards in the predicted impacts of climate change on human populations. Global Ecology and Biogeography. 2011;20(4):532–44. 10.1111/j.1466-8238.2010.00632.x

[34] Saraswat C, Kumar P. Climate justice in lieu of climate change: a sustainable approach to respond to the climate change injustice and an awakening of the environmental movement. Energy, Ecology and Environment. 2016;1(2):67–74. 10.1007/s40974-015-0001-8

[35] Liberman M, Trope Y, Stephan E. Psychological Distance. In: Kruglanski AW, Higgins ET, editors. Social Psychology: Handbook of Basic Principles. New York City: Guilford Press; 2007. p. 353.

[36] Spence A, Poortinga W, Pidgeon N. The Psychological Distance of Climate Change. Risk Analysis. 2012;32(6):957–72. 10.1111/j.1539-6924.2011.01695.x 21992607

[37] Jones C, Hine DW, Marks ADG. The Future is Now: Reducing Psychological Distance to Increase Public Engagement with Climate Change. Risk Analysis. 2017;37(2):331–41. 10.1111/risa.12601 26989845

[38] Loy LS, Spence A. Reducing, and bridging, the psychological distance of climate change. Journal of Environmental Psychology. 2020;(67):101388. 10.1016/j.jenvp.2020.101388

[39] Pahl S, Bauer J. Overcoming the Distance: Perspective Taking With Future Humans Improves Environmental Engagement. Environment and Behavior. 2013;45(2):155–69. 10.1177/0013916511417618

[40] McDonald RI, Chai HY, Newell BR. Personal experience and the “psychological distance” of climate change: An integrative review. Journal of Environmental Psychology. 2015;(44):109–18. 10.1016/j.jenvp.2015.10.003

[41] Kant I. Groundwork of the metaphysics of morals. Cambridge; 1785.

[42] Killen M, Smetana JG. Origins and development of morality. In: Lerner RM, editor. Handbook of child psychology and developmental science. Hoboken: John Wiley & Sons; 2015. p. 1–49.

[43] Turiel E. The development of social knowledge: Morality and convention. Cambridge: Cambridge University Press; 1983. 10.1159/000194532

[44] Aquino K, Reed A II, Thau S, Freeman D. A grotesque and dark beauty: How moral identity and mechanisms of moral disengagement influence cognitive and emotional reactions to war. Journal of Experimental Social Psychology. 2007;43(3):385–92. 10.1016/j.jesp.2006.05.013

[45] Darley JM, Latané B. Bystander Intervention in Emergencies: Diffusion of Responsibility. Journal of Personality and Social Psychology. 1968;8(4):377–83. 10.1037/h0025589 5645600

[46] Frantz CM, Mayer FS. The Emergency of Climate Change: Why Are We Failing to Take Action? Analyses of Social Issues and Public Policy. 2009;9(1):205–22. 10.1111/j.1530-2415.2009.01180.x

[47] Sommer M, Rucht D, Haunss S, Zajak S. Fridays for Future: Profil, Entstehung und Perspektiven der Protestbewegung in Deutschland [Internet]. Berlin: ipb working papers; 2019(2). Availabe from: https://www.otto-brenner-stiftung.de/fileadmin/user_data/stiftung/02_Wissenschaftsportal/03_Publikationen/2019_ipb_FridaysForFuture.pdf. German.

[48] Wahlström M, Kocyba P, De Vydt M, de Moor J, editors. Protest for a future: Composition, mobilization and motives of the participants in Fridays For Future climate protests on 15 March, 2019 in 13 European cities [Internet]. Staffordshire: Keele University; 2019. Available from: https://eprints.keele.ac.uk/6571/7/20190709_Protest%20for%20a%20future_GCS%20Descriptive%20Report.pdf.

[49] Koos S, Lauth F. Die Entwicklung und Zukunft der Fridays for Future-Bewegung: Ergebnisse von zwei Befragungen während der Fridays for Future-Demonstrationen in Konstanz am 24. Mai und 20. September 2019: Forschungsbericht. Konstanzer Online-Publikations-System. 2019 9;1–10. German.

[50] Newman KS, Jacobs ES, 2010. Who cares?: Public ambivalence and government activism from the New Deal to the second gilded age. Princeton: Princeton University Press; 2010.

[51] Pontes AI, Henn M, Griffiths MD. Assessing young people’s political engagement: A critical and systematic literature review of the instruments used to measure political engagement. International Politics Reviews. 2016;4(2):55–72. 10.1057/s41312-016-0002-4

[52] Winterich KP, Aquino K, Mittal V, Swartz R. When moral identity symbolization motivates prosocial behavior: The role of recognition and moral identity internalization. Journal of Applied Psychology. 2013;98(5):759–70. 10.1037/a0033177 23751218

[53] Doolittle A, Faul AC. Civic engagement scale: A validation study. SAGE Open. 2013;3(3):1–7. 10.1177/2158244013495542

[54] Böhm G, Pfister HR. Consequences, morality, and time in environmental risk evaluation. Journal of Risk Research. 2005;8(6):461–79. 10.1080/13669870500064143

[55] Fritsche I, Jonas E, Kayser DN, Koranyi N. Existential threat and compliance with pro-environmental norms. Journal of Environmental Psychology. 2010;30(1):67–79. 10.1016/j.jenvp.2009.08.007

[56] Marquit JD. Threat Perception as a Determinant of Pro- Environmental Behaviors: Public Involvement in Air Pollution Abatement in Cache Valley, Utah [M. Sc. thesis]. Logan, UT: Utah State University; 2008.

[57] Satow L. Skala zur Erfassung von Testverfälschung durch positive Selbstdarstellung und sozialerwünschte Antworttendenzen (SEA) [Internet]. Markdorf: Psychomeda Discussion Paper; 2012. Availabe from: www.psychomeda.de. German.

[58] Kavussanu M., Stanger N., & Ring C. (2015). The effects of moral identity on moral emotion and antisocial behavior in sport. Sport, Exercise, and Performance Psychology, 4(4), 268.

[59] Seguin C, Pelletier LG, Hunsley J. Toward a model of environmental activism. Environment and Behavior. 1998;30(5):628–52.

[60] Bierhoff HW, Wegge J, Bipp T, Kleinbeck U, Attig-Grabosch C, Schulz S. Entwicklung eines Fragebogens zur Messung von Eigenverantwortung oder:“Es gibt nichts Gutes, außer man tut es”. Zeitschrift für Personalpsychologie. 2005;4(1):4–18. 10.1026/1617-6391.4.1.4 German.

[61] R Core Team. R: A language and environment for statistical computing [software]. R Foundation for Statistical Computing. 2016. Available from: http://www.R-project.org/.

[62] Pena EA, Slate EH. Gvlma: Global validation of linear models assumptions. R package version 1.0.0.3 [software]. 2019. Available from: https://CRAN.R-project.org/package=gvlma. 10.1198/016214505000000637 PMC2820257 20157621

[63] Dietz T, Kalof L, Stern PC. Gender, values, and environmentalism. Social Science Quartlerly. 2002;83(1):353–64.

[64] Hunter LM, Hatch A, Johnson A. Cross-national gender variation in environmental behaviors. Social Science Quarterly. 2004;85(3):677–94. 10.1111/j.0038-4941.2004.00239.x

[65] McCright AM. The effects of gender on climate change knowledge and concern in the American public. Population and Environment. 2010;32(1):66–87. 10.1007/s11111-010-0113-1

[66] Vicente-Molina MA, Fernández-Sainz A, Izagirre-Olaizola J. Does gender make a difference in pro-environmental behavior? The case of the Basque Country University students. Journal of Cleaner Production. 2018;(176):89–98. 10.1016/j.jclepro.2017.12.079

[67] Zelezny LC, Chua PP, Aldrich C. Elaborating on gender differences in environmentalism. Journal of Social Issues. 2000;56(3):443–57. 10.1111/0022-4537.00177

[68] Finucane ML, Slovic P, Mertz CK, Flynn J, Satterfield TA. Gender, race, and perceived risk: The “white male” effect. Health, Risk and Society. 2000;2(2):159–72. 10.1080/713670162

[69] Flynn J, Slovic P, Mertz CK. Gender, Race, and Perception of Environmental Health Risks. Risk Analysis. 1994;14(6):1101–8. 10.1111/j.1539-6924.1994.tb00082.x 7846319

