# Supplementary material for: A question of morals? The role of moral identity in support of the youth climate movement Fridays4Future
Source: PLoS One. 2021 Mar 25;16(3):e0248353. doi: 10.1371/journal.pone.0248353 (PMC7993835; doi:10.1371/journal.pone.0248353)
Supplement: S1 Appendix — (DOCX) [file pone.0248353.s001.docx]

S1 Appendix

**Survey (English translation)**

**0. Introduction**

*Fridays for Future (F4F) is a worldwide movement of students and schoolchildren that is committed to climate protection. According to F4F's own statement, the school strike is about the failure of political leaders to address man-made climate change caused by greenhouse gas emissions. The consequences of climate change threaten not only animals and plants on the planet, but also the future of young people and future generations. Following the example of the initiator Greta Thunberg, pupils take to the streets and protest on Fridays during school hours. The protest takes place worldwide and is organized by the pupils and students themselves.*

*By now, several partner movements have been founded that support F4F, such as Parents for future, Artists for future, Together for future, etc.*

*In what follows we ask you to answer some questions regarding F4F. For the moment, these questions relate to F4F specifically and not to other organizations with similar goals (e.g., Greenpeace or NABU). We will specifically ask you about those in the end.*

**1. Attitudes (passive support)**

(1: I do not agree at all - 7: I completely agree)

1. *I like the F4F movement.*
2. *I do not like the actions of F4F. (R)*
3. *All people should join the actions of F4F.*
4. *I approve of the weekly demonstrations, even if they obstruct the traffic in the big cities.*
5. *I would like to support F4F.*
6. *Although they stay away from school, the students of F4F act right.*

**2. Active Support**

1. *Participation in F4F demonstrations (1: never before - 5: every Friday)*
2. *Signing petitions demanding the demands of F4F. (1: never -5: many)*
3. *Donations: (no: 0/yes: 1)*
4. *Amount of the donation (dropped)*
5. *Member of partner organisation: (no: 0/ yes: 1)*
6. *Strength of active involvement in a partner organisation (dropped)*
7. *other type of support (dropped)*

**3. Other pro-environmental behavior**

*Please estimate below your commitment to one or more other nature conservation organisations (such as Greenpeace, WWF, NABU, etc.):*

*(1: not at all - 4: very strong)*

1. *through membership*
2. *by participating in demonstrations*
3. *by public subscription*
4. *through volunteerism*

**4. Pro-environmental personal behavior**

*To what extent do the following statements apply to you?*

*(1: not at all - 4: very strongly)*

1. *I avoid flying and pay CO2 compensation if I have to fly.*
2. *I eat meat or sausage every day.*
3. *I avoid rubbish, for example by using returnable cups when I buy coffee to go.*
4. *I drive to work by car.*
5. *I make sure I buy environmentally friendly products when I shop.*

**5. Perception of climate change as a threat**

*How dramatic do you consider the consequences of climate change?*

*(1: not dramatic at all - 4: very dramatic)*
