# Supplementary material for: A question of morals? The role of moral identity in support of the youth climate movement Fridays4Future
Source: PLoS One. 2021 Mar 25;16(3):e0248353. doi: 10.1371/journal.pone.0248353 (PMC7993835; doi:10.1371/journal.pone.0248353)
Supplement: S2 Appendix — (DOCX) [file pone.0248353.s002.docx]

S2 Appendix

**Regression Tables**

Table B.1

*Regression coefficients: Model 1*

| Predictor | *b* | *Std. Error* | *t* | *p* | *bootstrapped*  95% CI | *R^2^* |
| --- | --- | --- | --- | --- | --- | --- |
| (Intercept) | 0.09 | 0.05 | 1.69 | .091 | [-0.00, 0.17] |  |
| MI Internalization | 0.13 | 0.05 | 2.86 | .004 | [0.03, 0.23] |  |
| MI Symbolization | 0.03 | 0.05 | 0.73 | .464 | [-0.07, 0.14] |  |
| Social Desirability | 0.02 | 0.04 | 0.45 | .650 | [-0.07, 0.11] |  |
| Age | -0.10 | 0.04 | -2.22 | .027 | [-0.19, -0.01] |  |
| Gender | -0.27 | 0.09 | -2.96 | .003 | [-0.46, -0.08] |  |
|  |  |  |  |  |  | .060** |

*Note:* * *p*<.05; ** *p*<.01; *** *p*<.001

Table B.2

*Regression coefficients: Model 2*

| Predictor | *b* | *Std. Error* | *t* | *p* | *bootstrapped*  95% CI | *R^2^* |
| --- | --- | --- | --- | --- | --- | --- |
| (Intercept) | 0.09 | 0.05 | 1.83 | .068 | [-0.00, 0.18] |  |
| MI Internalization | 0.15 | 0.05 | 3.11 | .002 | [0.04, 0.25] |  |
| MI Symbolization | 0.02 | 0.05 | 0.35 | .728 | [-0.08, 0.12] |  |
| Social Desirability | 0.01 | 0.04 | 0.27 | .790 | [-0.07, 0.11] |  |
| Age | -0.08 | 0.04 | -1.78 | .077 | [-0.17, 0.00] |  |
| Gender | -0.29 | 0.09 | -3.20 | .001 | [-0.49, -0.11] |  |
|  |  |  |  |  |  | .059** |

*Note:* * *p*<.05; ** *p*<.01; *** *p*<.001

Table B.3

*Regression coefficients: Model 3*

| Predictor | *b* | *Std. Error* | *t* | *p* | *bootstrapped*  95% CI | *R^2^* |
| --- | --- | --- | --- | --- | --- | --- |
| (Intercept) | -0.01 | 0.05 | -0.13 | .900 | [-0.10, 0.10] |  |
| MI Internalization | -0.02 | 0.05 | -0.33 | .741 | [-0.12, 0.07] |  |
| MI Symbolization | 0.13 | 0.05 | 2.78 | .006 | [0.03, 0.23] |  |
| Social Desirability | 0.06 | 0.04 | 1.39 | .166 | [-0.02, 0.14] |  |
| Age | -0.17 | 0.04 | -3.81 | < .001 | [-0.26, -0.08] |  |
| Gender | 0.02 | 0.09 | 0.23 | .822 | [-0.15, 0.20] |  |
|  |  |  |  |  |  | .044** |

*Note:* * *p*<.05; ** *p*<.01; *** *p*<.001

Table B.4

*Regression coefficients: Model 4*

| Predictor | *b* | *Std. Error* | *t* | *p* | *bootstrapped*  95% CI | *R^2^* |
| --- | --- | --- | --- | --- | --- | --- |
| (Intercept) | 0.04 | 0.05 | 0.80 | .427 | [-0.05, 0.12] |  |
| MI Symbolization | 0.11 | 0.04 | 2.77 | .006 | [0.03, 0.20] |  |
| Age | -0.05 | 0.04 | -1.21 | .227 | [-0.13, 0.03] |  |
| Risk Perception | 0.45 | 0.04 | 11.70 | < .001 | [0.36, 0.53] |  |
| Gender | -0.11 | 0.08 | -1.38 | .169 | [-0.29, 0.05] |  |
| Social Desirability | 0.05 | 0.04 | 1.26 | .209 | [-0.03, 0.13] |  |
|  |  |  |  |  |  | .248** |

*Note:* * *p*<.05; ** *p*<.01; *** *p*<.001
